# Enrichment of Polyglucosylated Isoflavones from Soybean Isoflavone Aglycones Using Optimized Amylosucrase Transglycosylation

**DOI:** 10.3390/molecules25010181

**Published:** 2020-01-01

**Authors:** Young Sung Jung, Ye-Jin Kim, Aaron Taehwan Kim, Davin Jang, Mi-Seon Kim, Dong-Ho Seo, Tae Gyu Nam, Chan-Su Rha, Cheon-Seok Park, Dae-Ok Kim

**Affiliations:** 1 Department of Food Science and Biotechnology, Kyung Hee University, Yongin 17104, Korea; chembio@khu.ac.kr (Y.S.J.); axk369@khu.ac.kr (A.T.K.);; 2 Graduate School of Biotechnology, Kyung Hee University, Yongin 17104, Korea; jinatiger@khu.ac.kr (Y.-J.K.); davin1031@khu.ac.kr (D.J.); cspark@khu.ac.kr (C.-S.P.); 3 Department of Food Science and Technology, Jeonbuk National University, Jeonju 54896, Korea; dhseo@jbnu.ac.kr; 4 Food Analysis Center, Korea Food Research Institute, Wanju 55365, Korea; ntg97@kfri.re.kr

**Keywords:** *Deinococcus geothermalis*, glycosyltransferase, *Glycine max* (L.) Merr., HPLC/MS, isoflavone aglycone-rich extract, isoflavone α-glucoside

## Abstract

Isoflavones in soybeans are well-known phytoestrogens. Soy isoflavones present in conjugated forms are converted to aglycone forms during processing and storage. Isoflavone aglycones (IFAs) of soybeans in human diets have poor solubility in water, resulting in low bioavailability and bioactivity. Enzyme-mediated glycosylation is an efficient and environmentally friendly way to modify the physicochemical properties of soy IFAs. In this study, we determined the optimal reaction conditions for *Deinococcus geothermalis* amylosucrase-mediated α-1,4 glycosylation of IFA-rich soybean extract to improve the bioaccessibility of IFAs. The conversion yields of soy IFAs were in decreasing order as follows: genistein > daidzein > glycitein. An enzyme quantity of 5 U and donor:acceptor ratios of 1000:1 (glycitein) and 400:1 (daidzein and genistein) resulted in high conversion yield (average 95.7%). These optimal reaction conditions for transglycosylation can be used to obtain transglycosylated IFA-rich functional ingredients from soybeans.

## 1. Introduction

Globally, soybean (*Glycine max* (L.) Merr.) is a large portion of major grain production, along with corn, wheat, and rice [1]. Isoflavones, a subgroup of flavonoids, are well-known plant secondary metabolites that are found mainly in the plant families *Fabaceae* or *Leguminosae*, including soybean [2,3]. Isoflavones in soybeans are present mainly in conjugated forms [4]. Soy isoflavones are classified as acetylisoflavones, malonylisoflavones, non-acylated isoflavone glucosides, and isoflavone aglycones (IFAs), depending on the side groups attached to the isoflavone skeleton [5]. IFAs have antioxidant properties, interact with epigenetic modifications, and exhibit estrogen-like activities [3]. Dietary IFAs can be metabolized to equol (4’,7-isoflavandiol), which has significant estrogenic activity in the intestine [6]. Soy isoflavones exist naturally in conjugated forms rather than as aglycones; for example, daidzein [7-hydroxy-3-(4-hydroxyphenyl)chromen-4-one], glycitein [7-hydroxy-3-(4-hydroxyphenyl)-6-methoxychromen-4-one], and genistein [5,7-dihydroxy-3-(4-hydroxyphenyl)chromen-4-one] [5]. However, conjugated isoflavones are converted into aglycone forms during processing or storage [4]. The bioaccessibility of IFAs is low due to their poor solubility in water [7]. Therefore, various methods have been developed to overcome these shortcomings of IFAs [8,9].

Glycosylation via chemical or non-chemical methods can change the physicochemical properties of compounds [10,11]. The use of enzymes, such as glycosyltransferases, is a natural way to improve the solubility of IFA [12]. Enzymatic glycosylation reactions are efficient and environmentally friendly [13]. Compared with harsh chemical methods, enzymatic methods typically generate five times less waste and have a 15-fold higher space–time yield [14]. Recently, biological glycosylation methods using a variety of enzymes to improve the water solubility of lipophilic compounds have been developed [11,15,16]. Transglycosylation improved genistein solubility by 3700–44,000 [17] and transglycosylated catechin, naringin, and rutin were found to have 100-, 1000-, and 30,000-fold higher water solubility than non-transglycosylated catechin, naringin, and rutin, respectively [18,19,20]. The use of glycosyltransferases from microbial sources for transglycosylation reactions is an efficient and eco-friendly biological alternative to chemical reactions [21]. Flavonoids can be glycosylated using enzymes from microbial sources such as cyclodextrin glycosyltransferase (CGTase) and uridine diphosphate glucose (UDP-glucose) [16,22,23,24]. CGTase is widely employed to increase the solubility and stability of polyphenols such as glycosylated isoflavones [23,25]. However, the CGTase-reacted complex solution is cloudy and contains high molecular weight compounds [26], while the UDP-glucose complex has low reaction yields and is expensive [16,27].

Amylosucrase (EC 2.4.1.4; AS) is a glycosyltransferase enzyme that belongs to the glucoside hydrolase family 13 [28]. One of the main characteristics of AS is that it catalyzes transglycosylation reactions to create α-1,4-glycosidic linkages given sucrose as a substrate [28,29]; AS promotes sucrose hydrolysis to release glucose and fructose, and α-1,4-oligosaccharides are formed using the released glucose as an acceptor [30]. *Deinococcus geothermalis* AS (DGAS) synthesizes α-1,4-glycosidic bonds using various acceptors such as flavonoids [31]. DGAS enhances the water solubility of flavonoid aglycones by transglycosylation [21,31,32]. Compared with glycosyltransferase enzymes such as CGTase, DGAS can react with high substrate specificity using relatively inexpensive donors [27]. In addition, the interpretation of experimental results is easier when using DGAS because of fewer impurities and the limited number of transglycosylated sugar moieties [27,32,33]. These features of DGAS can be exploited to increase the solubility and bioavailability of bioactive phenolic compounds in soybeans.

In this study, we investigated the physicochemical changes of major soybean IFAs in response to DGAS-mediated transglycosylation. To determine what reaction conditions resulted in high transglycosylated conversion yields of IFAs, factors such as the amount of donors, acceptors, and enzymes were evaluated. Optimal reaction conditions were applied to soybean isoflavone extract to confirm that the IFAs were efficiently transglycosylated. Based on our results, we propose an application of the DGAS enzyme process to enrich transglycosylated IFAs in soy-based foods such as soybeans, soybean extracts, and soy products in the industrial field.

## 2. Results and Discussion

### 2.1. DGAS Expression and Activities

**Figure 1:**
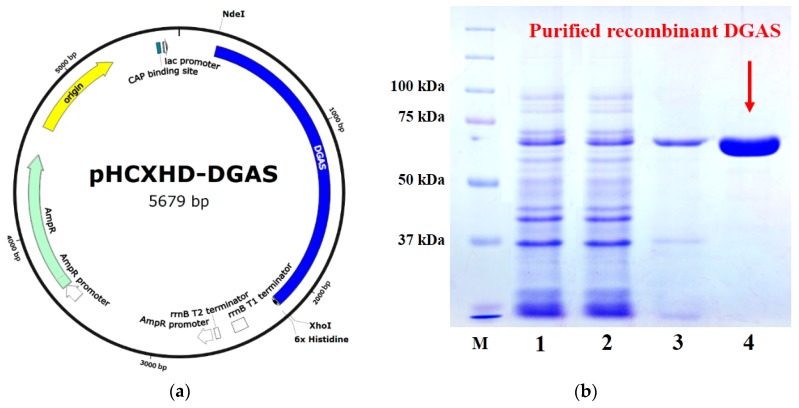
(**a**) Construction of expression vector of *Deinococcus geothermalis* amylosucrase (DGAS). (**b**) SDS-PAGE analysis of recombinant DGAS expressed in *Escherichia coli* and purified on a nickel– nitrilotriacetic acid affinity column. (Lane M, protein molecular standard marker; Lane 1, crude enzyme; Lane 2, crude passing through enzyme; Lane 3, inclusion body; Lane 4, purified enzyme).

DGAS was successfully expressed by *Escherichia coli* transformed with the shuttle vector pHCXHD-DGAS as confirmed by the detection of *dgas* expression in transformed cells (Figure 1a). Activity of AS was observed in cell extracts of pHCXHD-DGAS-transformed cells. DGAS fused with 6× histidine appeared as a single band with a molecular mass of about 73 kDa (Figure 1b), which is in good agreement with the estimated molecular mass of DGAS. This result confirmed that DGAS was successfully expressed as a functional protein in *E. coli*. DGAS was considered to be free of protein toxicity because it was not harmful to *E. coli*. Temperature and pH profiles of DGAS expressed in *E. coli* were investigated in the range of 30 to 55 °C and pH 4.0 to 9.0, respectively, based on a previous report of DGAS expression in *E. coli* [30]. It was previously reported that isoflavone glucosides can be thermally transformed to their corresponding aglycone forms during processing [34]. In consideration of the stability of the IFAs, the temperature and pH for the DGAS-mediated transglycosylation of IFAs in this study were set at 45 °C and pH 5.0, respectively.

### 2.2. Transglycosylation of IFAs with DGAS

**Figure 2:**
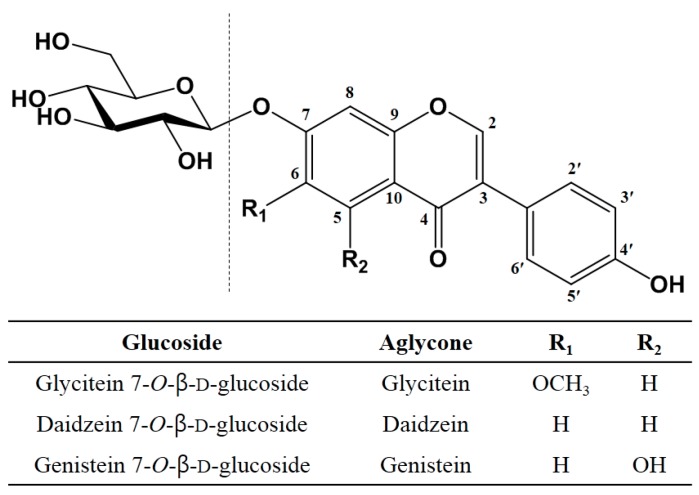
Structures of isoflavone glucosides (daidzin, glycitin, and genistin) present in soybeans.

Three IFAs (daidzein, glycitein, and genistein) are found in soybeans, and their glucosides are linked by β-glycosidic bonds at C-7 (Figure 2) [4]. Glycosylation carried out by glycosyltransferases, such as AS, generates two types of *O*-glucosides, which are commonly defined as α- and β-linked glucosides [35]. The detailed glycosylation mechanism has not been clearly elucidated, but the glycosylation reaction usually occurs on the anomeric carbon with the attack of weak nucleophiles, such as the −OH group of sugars [35]. Glycosylation reactions often involve a unimolecular (S_N_1) or bimolecular (S_N_2) nucleophilic substitution at the anomeric center [35]. 

**Figure 3:**
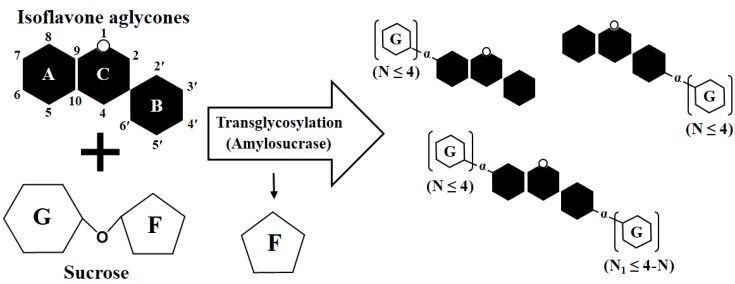
Scheme of transglycosylation reactions of isoflavone aglycones (IFAs) and sucrose using DGAS. G, glucose; F, fructose.

DGAS used in this study produced various transglycosylated isoflavones with α-1,4-glycosidic linkages from IFAs (Figure 3). The transglycosylation of IFAs (such as in genistein) using DGAS are likely to occur at the C-7 or C-4′ positions rather than the C-5 position. Previous studies reported that recombinant AS created various stereostructures of acceptor compounds due to position-specific transglycosylation [27,33] (Figure 3). Moreover, the transglycosylation of flavonoids using DGAS has been reported to occur primarily at the C-7 position of the A ring and at the C-4′ position of the B ring [27,32,36]. DGAS has been reported to attach four or fewer glucose moieties to flavonoids in transglycosylation [21,32]. Thus, we expected the total number of glucose moieties attached to IFAs in the DGAS transglycosylation process to be four. For example, if four glucoses bind to the C-7 position, the number of glucoses that can attach to the C-4′ position is zero, and if three glucoses bind to the C-4′ position, one additional glucose may bind to the C-7 position (Figure 3).

**Figure 4:**
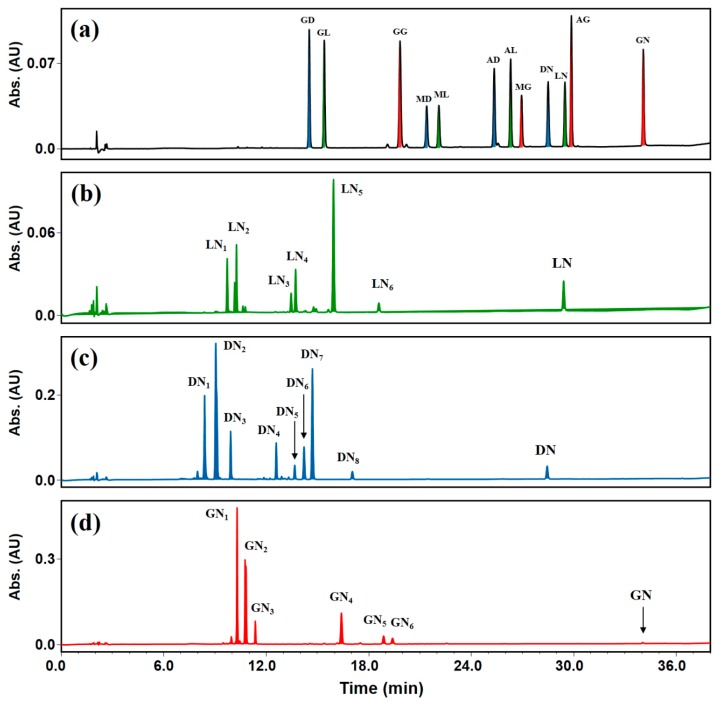
High-performance liquid chromatography (HPLC) traces (254 nm) of IFA standards after transglycosylation using DGAS. (**a**) Isoflavone standard mixture, (**b**) transglycosylated glycitein, (**c**) transglycosylated daidzein, and (**d**) transglycosylated genistein. Peaks: GD, daidzin; GL, glycitin; GG, genistin; MD, malonyldaidzin; ML, malonylglycitin; AD, acetyldaidzin; AL, acetylglycitin; MG, malonylgenistin; DN, daidzein; LN, glycitein; AG, acetylgenistin; GN, genistein. Peaks labeled LN_1_ to LN_6_, DN_1_ to DN_8_, and GN_1_ to GN_6_ are transglycosylated products from each IFA. Enzymatic transglycosylation reactions were performed using 1.0 mM of glycitein, 5.0 mM of daidzein, or 5.0 mM of genistein with 5.0 U of DGAS and 2.0 M of sucrose.

To identify reactants and products after transglycosylation, 12 isoflavone standards found in soybeans were separated by high-performance liquid chromatography (HPLC; peaks labeled: GD, daidzin; GL, glycitin; GG, genistin; MD, malonyldaidzin; ML, malonylglycitin; AD, acetyldaidzin; AL, acetylglycitin; MG, malonylgenistin; DN, daidzein; LN, glycitein; AG, acetylgenistin; GN, genistein) (Figure 4a). The results of transglycosylation using DGAS of the three IFA standards are shown chromatographically in Figure 4b–d. Compared to the 12 isoflavone standards, six to eight new peaks were detected, indicating that various transglycosylated products were produced from each aglycone during the enzymatic process (Figure 4b–d). Transglycosylated products from daidzein tended to be more polar (faster retention time) than transglycosylated glycitein and genistein glucosides for the same HPLC analysis conditions. This tendency was the same as the elution order of the three aglycones: daidzein > glycitein > genistein (Figure 4a). We attributed the different elution order of transglycosylated IFAs to their polarity differences, which are mainly influenced by the polarity of the parent compounds (acceptors in this study). If patterns of bound positions and the number of glucose moieties were the same in each aglycone, the elution order of transglycosylated IFAs according to increasing retention time would have been as follows: transglycosylated daidzein > transglycosylated glycitein > transglycosylated genistein. It was previously reported that α-glycosylisoquercitrin with more glucose moieties produced by the enzymatic transglycosylation of isoquercitrin eluted earlier than α-glycosylisoquercitrin with fewer glucose moieties [37]. Furthermore, after the transglycosylation of daidzin using DGAS, daidzein triglucoside eluted faster than daidzein diglucoside [36]. Therefore, in this study, transglycosylated IFAs with more glucose moieties had a faster elution time in reversed-phase HPLC analysis than transglycosylated IFAs with fewer glucose moieties (Figure 4b–d).

Peaks observed at the very beginning of elution (up to 12 min of retention time in this study) of the chromatograms were considered to be transglycosylated IFAs with two, three, or four glucose moieties (Figure 4b–d). However, more polar products (transglycosylated IFAs with 2–4 glucose moieties) among the transglycosylated IFAs resulted in lower resolution in the chromatogram. It was previously reported that transglycosylated products generated from the DGAS-catalyzed transglycosylation of isoquercitrin had similar retention times to each other, which resulted in very low resolution and greater difficultly separating them [32]. In addition, some isoquercitrin glucosides with high polarity have been reported to exist as isomers with the same number of sugars, the molecular weights of which were the same in mass spectra [32]. Based on previously reported results [32], we hypothesized that the transglycosylated IFA glucosides that eluted at similar retention times in this study were likely to be isomers with the same number of glucose moieties bound at different positions.

Peaks (except for minor peaks < 5% of the total product area) of transglycosylated reaction products from glycitein, daidzein, and genistein after enzymatic modification were labeled (Figure 4b–d). Peak LN_5_ in Figure 4b had a retention time similar to glycitin (glycitein 7-β-*O*-glucoside; peak GL of Figure 4a). The retention times of peak DN_7_ in Figure 4c and peak GN_6_ in Figure 4d were similar to those of daidzin (daidzein 7-β-*O*-glucoside; peak GD of Figure 4a) and genistin (genistein 7-β-*O*-glucoside; peak GG of Figure 4a), respectively. Thus, we assumed that peaks LN_5_, DN_7_, and GN_6_ were α-glucosides of IFA with one glucose moiety attached. Standard compounds (daidzin, glycitin, and genistin) found in soybeans have a β-glycosidic bond between IFA and glucose. When IFAs are transglycosylated using DGAS, glucose moieties bound to the −OH group at the C-7 or C-4′ position in IFAs are present in an α-glycosidic bond with IFAs [27,33]. The 7-α-monoglucosides (peaks LN_5_, DN_7_, and GN_6_) from the enzymatic modification of IFAs had different but similar retention times compared to standard isoflavone 7-β-monoglucosides (Figure 4b–d). Previously, daidzein triglucoside was reported to be more polar than daidzein diglucoside [36]. Due to their greater number of transglycosylated glucose moieties, LN_1_–LN_4_ from glycitein transglycosylation, DN_1_–DN_6_ from daidzein transglycosylation, and GN_1_–GN_5_ from genistein transglycosylation had higher polarities than LN_5_, DN_7_, and GN_6_, respectively (Figure 4b–d). DGAS produces higher amounts of flavonoid glucosides attached with a greater number of glucose molecules to parent flavonoids such as isoquercitrin and daidzin than glycosyltransferases such as CGTase and amyloglucosidase [27,32,36], which produce higher levels of flavonoid glucosides transglycosylated with fewer sugar moieties [22,24]. When UDP-glucose is used as a donor for glycosidic binding, only one or two new transglycosylated products are produced by glycosyltransferases [16,22]. In contrast, DGAS generated a variety of new transglycosylated products from the IFAs (Figure 4b–d). As shown in Figure 3, the number of transglycosylated glucoses has been reported to range from one to four [27,32], because the free hydroxyl (−OH) groups of flavonoid aglycones and their transglycosylated glucosides can be used as potential transglycosylation sites in the DGAS enzyme process.

### 2.3. Effects of Reaction Conditions on DGAS Transglycosylation

Table 1 shows the conversion yields of IFAs to transglycosylated IFA glucosides according to the concentrations of donor and acceptors as well as the amount of DGAS. Glycitein reacted at lower concentrations than daidzein and genistein because of its lower solubility [38]. Regardless of IFA and sucrose concentrations, the conversion yield of each IFA used in this study increased as the amount of DGAS used increased, whereas the conversion yield of each IFA generally decreased with increasing IFA concentration. In transglycosylation reactions, a large amount of enzymes ensures not only a fast reaction rate, but also an increased reaction yield [33]. However, at a low donor concentration (0.1 M) and high acceptor concentration (20 mM), the conversion yields for daidzein and genistein were the lowest during transglycosylation for the highest amount of enzymes (5.0 U). The ratio of donor to acceptor suggests that the conversion yield is affected even if a sufficient amount of enzyme is supplied. Similar to the results of this study (Table 1), previous studies reported that the conversion efficiency increased when the concentration of the donor was higher than that of the acceptor [31,39,40]. The enzyme-modified glycosylation of lipophilic compounds is known to be complex because of the low solubility of lipophilic compounds in aqueous systems [41]. To increase the conversion yield despite low solubility, various solvents such as ionic liquids have been used in enzyme reactions for flavonoid glucoside synthesis [13,41]. In this study, we used dimethyl sulfoxide (DMSO) as the solvent to dissolve all IFAs (acceptors). However, cloudiness was observed during the enzyme reaction when the amount of acceptor was increased in an aqueous system containing a donor, acceptor, buffer, and enzyme (data not shown). Acceptor solubility was found to be the most important factor determining the lowest conversion yields at the highest acceptor conditions (glycitein, 4 mM; daidzein and genistein, 20 mM) (Table 1). Therefore, it is reasonable to assume that the reason glycitein had the lowest conversion yield was its relatively lower solubility than that of daidzein and genistein.

The conversion yields of daidzein tended to be similar to those of genistein for the various reaction conditions used in this study. Glycitein had a lower conversion yield than genistein and daidzein (Table 1). Transglycosylation has been reported to occur at the hydroxyl group of the acceptor, resulting in the formation of an *O*-glycosidic bond between the acceptor and donor [35]. Likewise, a correlation between the number of hydroxyl groups and reactivity after the transglycosylation of hydroxyflavonoids with DGAS was observed [27]. The average conversion yields of daidzein and genistein were approximately 96.7% and 94.8%, respectively, at the highest amount of enzyme (5.0 U), lower acceptor concentrations (0.2–5.0 mM), and higher donor concentrations (1.0–2.0 M). In general, the highest conversion yield is achieved when sufficient enzymes and sucrose are provided for transglycosylation [33]. However, excess sucrose has been reported to reduce conversion yields [32]. Therefore, it is essential to have an adequate ratio of donor and acceptor as well as a sufficient amount of enzyme to attain higher conversion yields.

### 2.4. Application of DGAS to IFA-Rich Extract from Soybeans

**Figure 5:**
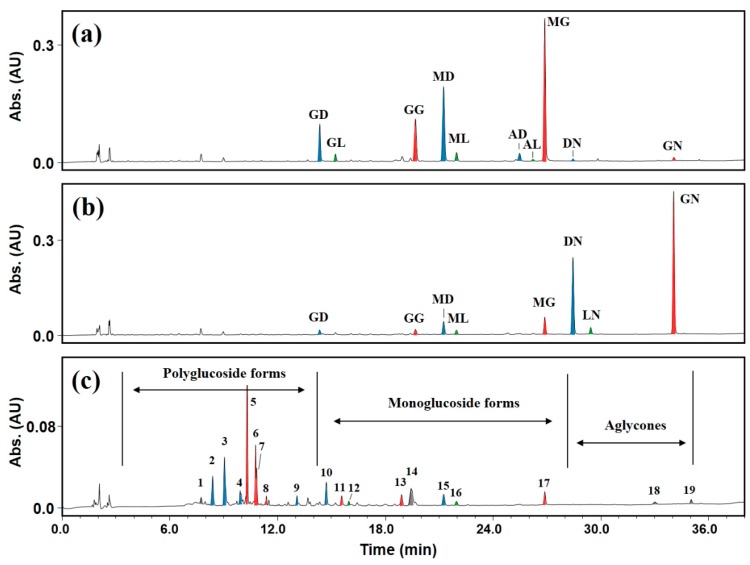
HPLC traces (254 nm) of IFA-rich soybean extract after transglycosylation using DGAS. (**a**) Soybean extract, (**b**) cellulase-treated soybean extract, (**c**) and cellulase-treated soybean extract transglycosylated using DGAS. Peaks: GD, daidzin; GL, glycitin; GG, genistin; MD, malonyldaidzin; ML, malonylglycitin; AD, acetyldaidzin; AL, acetylglycitin; MG, malonylgenistin; DN, daidzein; LN, glycitein; GN, genistein. Refer to Table 3 for the identification of each numbered peak of the 19 compounds.

Ultrasound-assisted extraction is known by its high efficiency, high productivity, lower consumption of solvents, and eco-friendly process [42]. In addition, in this study, to prevent protein interference after the extraction of soybeans with organic solvent, proteins were precipitated by dehydration and aggregation using ice-cold acetone [43]. After the successful conversion of IFAs into transglycosylated IFAs, optimal reaction conditions established for the DGAS process were used for the transglycosylation of IFA-rich soybean extract (SBE) as the acceptor. IFAs in SBE existed at lower concentrations than the other isoflavones such as isoflavone glucosides (Figure 5a and Table 2). 

Isoflavones in soybeans are mainly present in malonyl forms when extracted under normal conditions such as neutral solvent, ambient temperature, and short extraction time [44]. Similarly, malonyl forms were found to be the major isoflavones in SBE in this study (Figure 5a). Peaks labeled MD, ML, and MG are malonyldaidzin, malonylglycitin, and malonylgenistin, respectively (Figure 4a and Figure 5a,b). Therefore, 7-β-*O*-glucoses (with acetyl and malonyl moieties) bound to IFA should be hydrolyzed using cellulase (CE) prior to DGAS transglycosylation. Extraction under harsh conditions (low pH and high temperature) and microbial fermentation has been used previously to obtain IFAs from soybeans [44]. However, acid-modified extraction is not suitable for enzymatic glycosylation reactions and is not environmentally friendly. Conjugated isoflavones (malonylisoflavones, acetylisoflavones, and non-acylated isoflavone glucosides) are converted into their non-conjugated counterparts using enzymes such as glycosylase [45]. In this study, we obtained IFAs by removing β-1,4-linked glucose moiety from conjugated isoflavones using CE. After CE processing, malonylisoflavones were degraded into their corresponding aglycones. As the concentrations of conjugated daidzeins (peaks GD, MD, and AD), conjugated glyciteins (peaks GL, ML, and AL), and conjugated genisteins (peaks GG and MG) in SBE decreased (Figure 5a), the concentrations of their corresponding IFAs increased after CE treatment (Figure 5b). CE-treated soybean extract (CE-SBE; IFA-rich extract) had the highest content of genistein (53.9% of total), daidzein (42.2% of total), and glycitein (4.0% of total) compared with SBE and CE-SBE transglycosylated using DGAS (CE-SBE-DGAS) (Table 2). Consistent with the results of this study, the content of glycitein derivatives in soybeans and their processed products has been reported to be less than 5% of the total isoflavone content [5]. 

Soy isoflavones have different ultraviolet-visible spectra depending on their aglycones [3,46]. The peaks newly produced by DGAS-treated transglycosylation were tentatively confirmed by comparison with the patterns of ultraviolet-visible spectra of isoflavone standards and molecular weight (Table 3). As results of mass spectrometry (MS), 19 products including four unknown compounds of DGAS-treated CE-SBE were tentatively identified (Table 3). The malonylisoflavones in SBE were not changed by DGAS (peaks 15, 16, and 17 of Figure 5c) in this study. In contrast, IFAs were not detected in CE-SBE-DGAS (Figure 5c and Table 2), indicating that IFAs in CE-SBE are successfully transglycosylated using DGAS. In previous studies, DGAS has been reported to transglycosylate a sugar moiety to the −OH group of the flavonoid aglycone [27,30,31]. It is also reported that glucose moiety was transferred to the isoflavone monoglucosides such as daidzin [36]. 

Among the peaks newly generated using DGAS, the peaks 10, 12, and 13 were identified as daidzein, glycitein, and genistein monoglucosides with different stereospecificity and retention time from daidzin, glycitin, and genistin, respectively (Figure 5c and Table 3). The generated isoflavone monoglucosides can be seen to have a similar polarity compared to the isoflavone 7-β-*O*-glucosides (peaks GD, GL, and GG) in the SBE (Figure 5a). The MS results of CE-SBE-DGAS indicate that the more polar components, which are eluted earlier than isoflavone monoglucosides, are transglycosylated with two or more glucose moieties (Figure 5c and Table 3). The treatment of IFA standards and IFA-rich extract with DGAS resulted in higher amounts of isoflavone polyglucosides than isoflavone monoglucosides (Figure 4b–d and Figure 5c). As mentioned earlier, DGAS creates various transglycosylated isoflavones from IFAs. Studies of enzyme-modified flavonoids have reported binding of one or more glucose moieties to aglycones [24,27]. It has also been reported that glucose-added products, which are more polar than daidzin, are produced when transglycosylation is carried out using daidzin as the acceptor [36]. Hence, by using DGAS for the transglycosylation of CE-SBE, various isoflavone polyglucosides with two and three glucose moieties bound to their backbones, as well as isoflavone monoglucosides, can be generated.

## 3. Materials and Methods

### 3.1. Chemicals

Sucrose, formic acid, DMSO, and sodium phosphate were purchased from Sigma-Aldrich Co., LLC (St. Louis, MO, USA). Daidzin, glycitin, genistin, daidzein, glycitein, and genistein were bought from Extrasynthese (Genay, France). Malonyldaidzin, malonylglycitin, malonylgenistin, acetyldaidzin, acetylglycitin, and acetylgenistin were purchased from Wako Chemical Co. (Tokyo, Japan). HPLC-grade water, acetonitrile, and methanol were obtained from Fisher Scientific (Hampton, NH, USA). All other chemicals used in this study were of analytical reagent grade.

### 3.2. Expression and Activity of DGAS

The *dgas* gene has been described in previous studies [21,30]. This gene was identified in the genome of *D. geothermalis* DSM 11300. *E. coli* MC1061 was used to express *dgas*, whereas *E. coli* DH10B was employed for gene manipulation. DGAS was expressed by *E. coli* MC1061 harboring *dgas* in the pHCXHD vector system, as described previously [32,36]. *Escherichia coli* transformed with pHCXHD-DGAS were incubated for 18 h and then harvested by centrifugation (Hanil Combi 514R; Hanil Centrifuge Co., Gimpo, Korea) at 7000× *g* for 20 min. Then, the supernatant was discarded, and the pellet was resuspended in lysis buffer (50 mM NaH_2_PO_4_, 300 mM NaCl, and 10 mM imidazole; pH 7.5) and disrupted using an iced ultrasonic bath (Sonifier 450; Branson Ultrasonics Corp., Danbury, CT, USA). Proteins extracted from the solution were collected by centrifugation (Hanil Combi 514R; Hanil Centrifuge Co.) at 7000× *g* at 4 °C for 20 min. Enzymes in the crude cell extract were purified with a nickel–nitrilotriacetic acid affinity column (Poly-Prep; Bio-Rad Laboratories, Inc., Hercules, CA, USA) filled with 500 μL nickel–nitrilotriacetic acid Superflow (Qiagen, Hilden, Germany). A purified enzyme was confirmed by performing sodium dodecyl sulfate–polyacrylamide gel electrophoresis using 10% (*v*/*v*) acrylamide. Protein concentrations were measured using a bicinchoninic acid assay kit (Thermo Fisher Scientific, Agawam, MA, USA) with bovine serum albumin as a standard.

The sucrose hydrolysis activity of DGAS was measured using 3,5-dinitrosalicylic acid solution, as described previously [32,36]. A reaction mixture comprising 50 μL 500 mM Tris-HCl (pH 8.0), 100 μL 25% (*w*/*v*) sucrose, and 300 μL deionized water was used. The enzymatic reaction was carried out by adding 50 μL of DGAS solution to the reaction mixture at 45 °C for 10 min. To stop the reaction, 500 μL of 3,5-dinitrosalicylic acid solution was added. After boiling for 5 min, the absorbance of the final reaction mixture was measured at 550 nm using a microplate reader (iMark™ Microplate Absorbance Reader; Bio-Rad Laboratories, Inc.). The reducing sugar concentration was calculated using a fructose standard curve. One unit of DGAS was defined as the amount of enzyme that produced one μmol of fructose per min under the assay conditions.

### 3.3. Transglycosylation of IFAs

Transglycosylation reactions with DGAS were performed using sucrose as the donor in sterilized water and IFA as an acceptor in DMSO. The reaction was performed in sodium phosphate buffer (pH 5.0 and 50 mM final concentration). Sucrose was used at concentrations of 10, 100, and 200 mM. Daidzein and genistein were used at concentrations of 20, 500, and 2000 μM, while glycitein was used at concentrations of 4, 100, and 400 μM. The amount of DGAS used was set to three enzyme units at 0.1, 1.0, and 5.0 U. The reaction temperature was 45 °C, and the reaction time was 24 h. These reaction conditions (temperature, buffer, and pH) for DGAS were based on those reported in previous studies [31,36]. The reaction was stopped by adding an equal amount of 10% (*v*/*v*) DMSO in methanol containing 0.1% (*v*/*v*) formic acid. The terminated reaction solution was subjected to vigorous mixing, sonicated for 10 min, and filtered through a 0.45 μm polyvinylidene fluoride syringe filter (GH Polypro; Pall Corp., Port Washington, NY, USA). Final samples were stored in the freezer at −20 °C prior to analysis.

### 3.4. Preparation and Transglycosylation of SBE

Soybean seeds (cv. Daewon) were obtained from the Department of Southern Area Crop Science, National Institute of Crop Science, Rural Development Administration (Dalseong, Korea). Soybean seeds (10 g) were pulverized using a cutting mill (Tube Mill 100 control; IKA^®^, Staufen, Germany). Extraction was carried out with 250 mL of 70% (*v*/*v*) aqueous ethanol using an ultrasonic bath (Sonifier 450; Branson Ultrasonics Corp.) at ambient temperature for 1 h. The aqueous ethanol extract of soybean seeds was filtered through Whatman no. 4 filter paper (Whatman Inc., Clifton, NJ, USA). The filtrate was evaporated using a rotary evaporator (N-1000; Eyela, Tokyo, Japan) in a water bath at 40 °C. Five hundred milliliters acetone was added to dry SBE, and this mixture was placed at −20 °C for 24 h. The mixture was filtered through Whatman no. 4 filter paper (Whatman Inc.) and evaporated (N-1000; Eyela). To obtain IFA-rich extract by enzymatically hydrolyzing conjugated isoflavones in SBE, 200 mg of SBE powder and 20 mg of CE (EC 3.2.1.6) (Vision Corp., Seoul, Korea) were reacted in water at 40 °C for 24 h. The reactant from the CE-SBE was concentrated in the methanol phase by removing the matrix using an ODS Sep-Pak cartridge (Oasis HLB; Waters, Milford, MA, USA). Purified CE-SBE reactant was evaporated using a rotary evaporator (N-1000; Eyela) in a water bath at 40 °C. For transglycosylation, CE-SBE powder (10 g/L) was used as an acceptor, and sucrose (100 mM) was used as the donor. The reaction temperature, time, buffer, and pH conditions of SBE were the same as those described in Section 3.3. After termination of the enzyme reaction, the pretreatment process for HPLC analysis proceeded in the same manner described in Section 3.3. The final samples were stored in a freezer at −20 °C until further analysis. 

### 3.5. Analysis of Transglycosylated Isoflavones by HPLC and MS

SBE, CE-SBE, and CE-SBE-DGAS were analyzed using a Waters HPLC system (Alliance e2998; Waters) equipped with a ProntoSIL ace-EPS-C_18_ column (120 Å, 5 μm, 4.6 × 250 mm; Bischoff, Leonberg, Germany) and a photodiode array detector (2695; Waters) at 254 nm. Gradient elution was performing using 0.1% (*v*/*v*) formic acid in water (solvent A) and acetonitrile (solvent B). All solvents were filtered, degassed, and kept under pressure. The initial mobile phase was 100% solvent A. The gradient of mobile phase B was as follows: 0–2.5 min, 0% B; 2.5–5 min, 0–12% B; 5–7 min, 12–18% B; 7–10 min, 18–18% B; 10–13 min, 18–26% B; 13–19 min, 26–26% B; 19–23 min, 26–46% B; 23–26 min, 46–46% B; 26–30 min, 46–75% B; 30–32 min, 75–0% B; 32–35 min, 0% B. Flow rate, column oven temperature, and injection volume were 1.0 mL/min, 30 °C, and 5 μL, respectively. 

The mass detection was measured using the modified method used in our previous study [36]. The quadrupole Dalton-based (QDa) detector (Waters) was used to obtain the MS data. An isocratic solvent manager system split the analyte phase into acetonitrile at a ratio of 8:2. The QDa parameters in positive ion mode were as follows: capillary voltage, 0.8 kV; cone voltage, 5 V; source temperature, 600 °C; desolvation gas flow, 800 L/h. The mass values to identify the transglycosylated isoflavones were used for daidzein glucosides (255.1, 417.1, 579.1, 741.1, and 903.1 *m*/*z*), glycitein glucosides (285.1, 447.1, 609.1, 771.1, and 933.1 *m*/*z*), and genistein glucosides (271.2, 433.2, 595.2, 757.2, and 919.2 *m*/*z*), respectively. Empower 3 (Waters) was used to control the HPLC-QDa system and analyze the data obtained.

### 3.6. Statistical Analysis

Data are displayed as means ± standard deviations of three replicate determinations. Statistical analyses were performed using IBM SPSS software version 23.0 (IBM SPSS Statistics Inc., Chicago, IL, USA). One-way analysis of variance was performed to assess the significance of differences in mean values. Significant differences were verified by the Tukey–Kramer honestly significant difference test (*p* < 0.05).

## 4. Conclusions

Using the DGAS enzymatic process, IFAs were modified to generate various forms of isoflavone glucosides with different polarities. In addition, transglycosylated IFAs had α-glycosidic bonds in the isoflavone backbone because of the enzymatic properties of DGAS. Different conversion yields of transglycosylation products were achieved for different reaction conditions. The most efficient transglycosylation resulted from a low acceptor concentration (IFAs), donor-high concentration (sucrose), and high DGAS enzyme activity. Applying the reaction conditions used in this study, IFA-rich extracts from SBE were obtained by an environmentally friendly process using CE. The treatment of IFA standards and IFA-rich extract with DGAS produced higher amounts of isoflavone polyglucosides than isoflavone monoglucosides. Taken together, the results of this study suggest that DGAS-treated transglycosylation changes physicochemical properties such as the bioactivity, solubility and stability of isoflavones in soybeans and produces soybean-based functional ingredients rich in transglycosylated IFA.

## Figures and Tables

**Table 1:** Conversion yields of soy isoflavone aglycones after *Deinococcus geothermalis* amylosucrase (DGAS)-treated transglycosylation according to concentrations of donor and acceptors and amounts of the DGAS enzyme.

DGAS (Units)	Sucrose (M)	Glycitein (mM)			Daidzein (mM)			Genistein (mM)		
		0.05	1.0	4.0	0.2	5.0	20	0.2	5.0	20
0.5	0.1	0.0 ± 0.0 ^c 1^	1.9 ± 1.7 ^c^	3.9 ± 0.2 ^b^	0.0 ± 0.0 ^f^	4.3 ± 0.6 ^de^	2.4 ± 0.4 ^ef^	10.9 ± 2.3 ^c^	3.1 ± 1.3 ^de^	2.1 ± 0.1 ^e^
	1.0	0.0 ± 0.0 ^c^	6.8 ± 0.8 ^a^	4.2 ± 0.2 ^b^	20.0 ± 2.5 ^b^	8.3 ± 1.0 ^cd^	2.7 ± 0.1 ^ef^	59.8 ± 0.4 ^b^	5.3 ± 0.3 ^d^	0.8 ± 0.0 ^e^
	2.0	0.0 ± 0.0 ^c^	6.5 ± 0.8 ^a^	4.9 ± 0.4 ^ab^	28.8 ± 3.0 ^a^	9.2 ± 1.0 ^c^	1.8 ± 0.2 ^ef^	68.6 ± 1.0 ^a^	3.5 ± 0.1 ^de^	1.2 ± 0.0 ^e^
1.0	0.1	0.0 ± 0.0 ^d^	7.0 ± 0.2 ^c^	6.0 ± 0.3 ^c^	22.5 ± 3.2 ^d^	12.0 ± 0.9 ^e^	4.4 ± 1.1 ^f^	19.3 ± 1.9 ^d^	7.1 ± 0.6 ^e^	1.3 ± 0.0 ^e^
	1.0	13.3 ± 2.5 ^b^	19.0 ± 1.7 ^a^	8.8 ± 0.4 ^bc^	50.0 ± 2.4 ^b^	32.5 ± 0.7 ^c^	6.9 ± 0.1 ^f^	53.1 ± 4.2 ^b^	28.5 ± 0.2 ^c^	4.8 ± 0.0 ^e^
	2.0	8.6 ± 3.6 ^bc^	20.8 ± 0.4 ^a^	23.2 ± 3.7 ^a^	55.5 ± 2.2 ^a^	32.4 ± 0.8 ^c^	4.8 ± 0.2 ^f^	62.5 ± 4.7 ^a^	24.7 ± 0.2 ^cd^	2.9 ± 0.2 ^e^
5.0	0.1	20.8 ± 6.4 ^e^	35.0 ± 1.6 ^d^	28.2 ± 0.8 ^de^	72.0 ± 1.5 ^b^	65.0 ± 0.8 ^c^	23.3 ± 0.3 ^f^	77.3 ± 2.7 ^b^	74.8 ± 0.5 ^bc^	23.4 ± 0.0 ^e^
	1.0	16.3 ± 1.5 ^e^	69.5 ± 9.2 ^b^	56.8 ± 2.2 ^c^	97.2 ± 2.8 ^a^	95.0 ± 0.1 ^a^	50.5 ± 0.0 ^d^	92.2 ± 5.1 ^a^	96.6 ± 1.0 ^a^	66.0 ± 0.2 ^d^
	2.0	18.0 ± 2.8 ^e^	88.8 ± 3.8 ^a^	52.1 ± 1.3 ^c^	97.5 ± 0.7 ^a^	96.9 ± 0.2 ^a^	36.9 ± 0.1 ^f^	92.1 ± 3.2 ^a^	98.2 ± 0.1 ^a^	70.1 ± 0.1 ^cd^

^1^ Data are expressed as means ± standard deviations (*n* = 3). Means with different superscripted letters indicate that for the same amount of DGAS, each isoflavone aglycone differed significantly by the Tukey–Kramer honestly significant difference test (*p* < 0.05).

**Table 2:** Content (mg/g powder) of isoflavones in soybean extract (SBE), cellulase (CE)-treated SBE (CE-SBE; isoflavone aglycone-rich extract), and CE-SBE transglycosylated using DGAS (CE-SBE-DGAS).

Isoflavone	SBE	CE-SBE	CE-SBE-DGAS
Glycitein	0.0 ± 0.0 ^b 1^	3.98 ± 0.2 ^a^	0.0 ± 0.0 ^b^
Daidzein	0.79 ± 0.13 ^b^	41.98 ± 1.26 ^a^	0.0 ± 0.0 ^c^
Genistein	1.0 ± 0.03 ^b^	53.63 ± 4.35 ^a^	0.0 ± 0.0 ^c^

^1^ Data are expressed as means ± standard deviations (*n* = 3). Means with different superscripted letters in the same row differ significantly by the Tukey-Kramer honestly significant difference test (*p* < 0.05).

**Table 3:** Identification of isoflavone derivatives of CE-SBE-DGAS.

Peak No.	Time (min)	λ_max_ (nm)	[M]^+^ (*m*/*z*)	Aglycone	Identified Compound
1	7.74	219.6, 276.6	n.d ^1^	Unknown	Unknown
2	8.38	248.1, 296.5	903.1	Daidzein	Daidzein tetraglucoside
3	9.04	248.1, 298.1	741.1	Daidzein	Daidzein triglucoside
4	9.91	248.1, 298.1	741.1	Daidzein	Daidzein triglucoside
5	10.30	257.1, 322.0	757.2	Genistein	Genistein triglucoside
6	10.77	257.1, 322.0	757.2	Genistein	Genistein triglucoside
7	10.83	257.6, 322.0	757.2	Genistein	Genistein triglucoside
8	11.38	257.6, 329.2	595.2	Genistein	Genistein diglucoside
9	13.08	248.1, 298.1	417.1	Daidzein	Daidzein diglucoside
10	14.77	245.7, 300.5	417.1	Daidzein	Daidzein monoglucoside
11	15.57	257.6, 322.0	595.2	Genistein	Genistein diglucoside
12	15.97	252.8, 319.6	447.1	Glycitein	Glycitein monoglucoside
13	18.90	257.6, 322.0	433.1	Genistein	Genistein monoglucoside
14	19.45	229.1, 271.8	n.d	Unknown	Unknown
15	21.25	248.1, 298.1	n.d	Daidzein	Malonyldaidzin
16	21.97	257.6, 317.2	n.d	Glycitein	Malonylglycitin
17	26.88	257.6, 324.4	n.d	Genistein	Malonylgenistin
18	33.02	267.1	n.d	Unknown	Unknown
19	35.04	233.8, 276.6	n.d	Unknown	Unknown

^1^ n.d Out of the set molecular weight in mass conditions.

## Abbreviations

AS	amylosucrase
CE	cellulase
CE-SBE	cellulase-treated soybean extract
CE-SBE-DGAS	CE-SBE transglycosylated using DGAS
CGTase	cyclodextrin glycosyltransferase
DGAS	*Deinococcus geothermalis* amylosucrase
DMSO	dimethyl sulfoxide
HPLC	high-performance liquid chromatography
IFA	isoflavone aglycone
MS	mass spectrometry
SBE	soybean extract
QDa	quadrupole Dalton-based
UDP-glucose	uridine diphosphate glucose
